# Anatomical networks reveal the musculoskeletal modularity of the human head

**DOI:** 10.1038/srep08298

**Published:** 2015-02-06

**Authors:** Borja Esteve-Altava, Rui Diogo, Christopher Smith, Julia C. Boughner, Diego Rasskin-Gutman

**Affiliations:** 1 Theoretical Biology Research Group, Cavanilles Institute of Biodiversity and Evolutionary Biology, University of Valencia, 46071 Valencia, Spain; 2 Department of Anatomy, Howard Univ. College of Medicine, Washington, DC, USA; 3 Department of Anatomy and Cell Biology, Univ. of Saskatchewan, Saskatoon, SK, Canada

## Abstract

Mosaic evolution is a key mechanism that promotes robustness and evolvability in living beings. For the human head, to have a modular organization would imply that each phenotypic module could grow and function semi-independently. Delimiting the boundaries of head modules, and even assessing their existence, is essential to understand human evolution. Here we provide the first study of the human head using anatomical network analysis (AnNA), offering the most complete overview of the modularity of the head to date. Our analysis integrates the many biological dependences that tie hard and soft tissues together, arising as a consequence of development, growth, stresses and loads, and motion. We created an anatomical network model of the human head, where nodes represent anatomical units and links represent their physical articulations. The analysis of the human head network uncovers the presence of 10 musculoskeletal modules, deep-rooted in these biological dependences, of developmental and evolutionary significance. In sum, this study uncovers new anatomical and functional modules of the human head using a novel quantitative method that enables a more comprehensive understanding of the evolutionary anatomy of our lineage, including the evolution of facial expression and facial asymmetry.

Evolution proceeds by innovating and, mostly, tinkering with available structures^1^. If these structures are too tightly intertwined, then slight changes in any of them may prove deleterious to the form and/or function of the organism. A way around this risk is modular organization, which allows each structure to evolve semi-independently and promotes evolvability^2^. Discovering phenotypic modules is thus a crucial task in evolutionary developmental biology (EvoDevo), helping not only to understand the evolution of organismal form^3^ but also to decipher the genotype-to-phenotype map^2^. Network analysis is a mathematical approach widely used to inspect intricate biological systems such as gene-regulatory networks, the brain, and food webs^4^. Network analysis also provides tools to identify the modules that compose these complex systems^5^. However, EvoDevo has seldom applied the power of network tools to investigate how modular is, and which specific modules form, our own body plan.

The human head is a particularly challenging body part to “unpack” because it encloses a dense physical organization of morphofunctional units with often-overlapping functions. While modularity analysis of the human head has come a long way in recent decades, all studies have focused so tightly on very specific biological relations that the identification of broad phenotypic modules has been hampered. Contemporary studies of phenotypic modularity focus mainly on functional and developmental analysis of skeletal units^6^,^7^,^8^. For example, cell origin or ossification-timing relations define developmental modules of bones^8^, while size and shape covariation among skeletal regions defines variational modules^7^. Analyzing the organization of bone arrangements in the skull using network tools has also proved useful to delimit quantitatively phenotypic modules in the human skull, thus helping to understand its evolutionary and developmental constraints^9^,^10^.

However, this emphasis on hard tissues tends to omit the role of soft tissues in shaping the modular organization of the head, which facilitates its stability, performance, and evolvability^11^. The Functional Matrix Hypothesis proposed by Moss^12^ was an early attempt to solve this enduring problem. In Moss' hypothesis, the head is divided into ‘functional components’ (i.e. modules) determined by soft tissues and cranial cavities (e.g. neural, ocular and oral), and bounded by the surrounding skeletal units: the soft tissue components guiding the development of the skeletal units. For instance, the temporalis, masseter and medial pterygoid muscles are said to form a functional component with the mandible because they help shape the dentary bone's form^13^. However, in most cases ‘functional components’ are merely informed conjectures based on assumptions about position, form and function that reflect *a priori* expectations rather than the results of quantitative analysis^14^,^15^. For example, a recent anatomical network analysis (AnNA) showed that functional matrices are essential to generate a proper pattern of connectivity of the face, whereas the pressure of the growing brain against the skull vault is not necessary to explain the connectivity pattern among the bones of the cranial vault and base^15^.

Here we use this new quantitative and objective approach (AnNA) to treat the skeletal, cartilaginous, and muscular units of the human head as the elements of a network (nodes), whose interactions at their physical contacts (links) determine the boundaries of the phenotypic modules of the head (Fig. 1 and Table 1; see Methods and SI for further details). Our driving hypothesis was that we should be able to (1) define phenotypic modules that reflect developmental, functional, and morphological aspects of the anatomy of the head, and thus (2) identify at least some modules that differ from those that were predicted purely by *a priori* theoretical or qualitative assumptions. Using AnNA also allowed us to analyze bone dependences in isolation from muscle dependences to further enrich our understanding of human head modularity.

Our study revealed that the *musculoskeletal network* of the adult human head and neck comprises 181 morphofunctional units (bones, cartilages and muscles) connected by 412 physical contacts. The head divides into 10 musculoskeletal modules that form coherent anatomical, functional, evolutionary and/or developmental complexes, which have never been suggested in the past (Fig. 1 and Table 1). Thus, studying general biological dependences using this well-defined quantitative method (AnNA) reveals unique insights about human head complexity–specifically its development, evolutionary origins and diseases–that are not readily apparent using conventional approaches.

The *lower jaw/inner ear musculoskeletal complex* (module 1) is particularly interesting because it groups morphofunctional units that would intuitively seem independent from each other given their anatomical contacts. Remarkably, AnNA highlights an unexpected, deeper connection: these units are linked by structures that share a major common developmental denominator, the first pharyngeal arch. For instance, this module comprises neurocranial bones and facial muscles of the ear region (e.g. auricularis posterior), which are not themselves derived from the first arch but contact a bone (malleus) and a muscle (tensor tympani) that are first arch derivatives. The skull area surrounding the ear region is in turn connected to the lower jaw by first arch muscles, such as the masseter, temporalis, pterygoideus lateralis, and digastricus anterior (via the digastricus posterior, which is a second arch muscle). Further, the mandible is connected via other first arch muscles (e.g. mylohyoideus) to the hyoid bone, which is a second arch structure; and thus also to the tongue, infrahyoid muscles, and some pharyngeal muscles. Evolutionarily, this musculoskeletal complex is particularly interesting because it reveals an intricate interplay between an ancient relationship in mammals (i.e. the lower jaw and inner ear bones) and various muscles that originated before the rise of mammals (e.g. genioglossus, geniohyoideus), alongside masticatory muscles with clear non-mammalian homologues^16^,^17^.

The *mid/upper face musculoskeletal complex* (module 2), which groups upper facial bones and muscles, illustrates how AnNA can coherently synthesize data from different sources (i.e. origin, growth, and function) to detect phenotypic modules not predicted using theoretical assumptions. As explained above, in Moss' model, the temporalis, masseter, and medial pterygoid muscles were grouped into a single module. However, AnNA groups the medial pterygoid muscle in the mid/upper face musculoskeletal module, and the masseter with the temporalis in the lower jaw/inner ear musculoskeletal module. Significantly, studies of human development pathologies (e.g. cleft lip and palate) have consistently shown a strong developmental and functional relationship between the upper and mid-face muscles salient to facial expression and musculoskeletal units related to palate movements^18^. Our results further support the idea that integrating muscle as well as skeletal modules yields new and deeper insights relevant in evolutionary developmental and medical contexts^10^.

The *laryngeal musculoskeletal complex* (module 3) constitutes a well-defined phenotypic module that includes the laryngeal cartilages and the muscles directly attaching these cartilages. The *neck musculoskeletal complex* (module 4) includes all the neck muscles innervated by cranial nerves that attach the skull to the nearby postcranial bones (i.e. cervical vertebrae, clavicles, scapulae, and sternum). This neck module is interesting as it groups muscles and bones with completely different developmental and evolutionary origins, indicating that this module is mainly defined by function. The *left and right oral/ocular complexes* (modules 5 and 6) group the maxillae and the zygomatic bones with orofacial muscles (see the *left and right orofacial muscular complexes* described below) together with the zygomaticus minor (orofacial), the depressor supercilii (ocular), and the inferior oblique (extrinsic) muscles. The *left and right superficial ear complexes* (modules 7 and 8) and the *left and right inner ear complexes* (modules 9 and 10) are also coherent functional modules: the former include only facial muscles related to the movements of the ear, the latter include only inner musculoskeletal structures of the inner ear. It is interesting to note that in the network analysis including only muscles the zygomaticus minor – an elevator of the upper lip – is not included in the orofacial muscle module with the zygomaticus major, while these two muscles are grouped in a same module in the network analysis including both muscles and the skeleton, as would be expected *a priori* based on function.

Importantly, the use of AnNA also allows one to efficiently separate the musculoskeletal network into its two main component networks–one skeletal and one muscular—thus facilitating the independent analysis of hard and soft morphofunctional units. The *skeletal network* comprises 45 bones and cartilages articulated at 86 contact surfaces (sutures, synchondroses, and synovial joints). This skeletal network divides into eight modules, which are shown in Fig. 2 and in Table 1. Among these eight well-delimited modules are a *cranial* (neurocranium and basicranium) and a *facial* (viscerocranium) *complex* as previous studies have reported^9^, thus indicating that AnNA can detect and further validate accepted modules. A further strength of the present work is that it is the first AnNA study to also include the mandible, the ear ossicles, the hyoid bone, and the laryngeal cartilages. By doing this, this study reveals that the cranial module includes the mandible with the bones of the vault and cranial base, because of the mandible's structural relation with the temporal bones (e.g. glenoid fossa); in contrast, the *left and right ossicles complexes* group auditory ossicles independently from other bones. The *thyroid complex* groups all laryngeal cartilages, while the hyoid bone forms its own module (by not including muscles in the skeletal networks, the hyoid bone is not connected directly to others skeletal structures). We included vertebrae, sternum, scapulae, and clavicles in our analysis because these bones also connect with head muscles (e.g. trapezius, sternocleidomastoideus, platysma): AnNA grouped them in two separate modules, the *thoracic* and the *cervical complexes*, because they are isolated by the absence of muscular attachments.

In turn, the *muscular network* comprises 136 muscles sparsely connected at 78 contact points (fiber fusions and well-defined tendons), and divides into three major modules and 21 smaller blocks of 4 to 2 muscles each. The three main modules are shown in Fig. 3 and in Table 1: a single *ocular/upper face complex*, and *left and right orofacial complexes*. It is remarkable that the three main muscular modules include muscles of facial expression exclusively. Recent comparative studies of primates have shown that facial expression muscles have undergone more evolutionary change (e.g. in shape, in appearance and loss, and in insertion shifting) than most other groups of head muscles during human evolution^17^,^19^,^20^. In addition, the evolution of facial muscles has been crucial to our particular abilities for verbal and visual communication^21^. Interestingly, none of these major and minor muscular complexes derive from a shared ontogenetic anlage, or a homogeneous developmental origin. For instance, some modules group a muscle of the 1^st^ arch (e.g. digastricus anterior) with muscles of the 2^nd^ arch (e.g. digastricus posterior, stylohyoideus) rather than other muscles of the 1^st^ arch. Instead, and importantly, muscular modules are functional complexes that integrate muscles with completely different phylogenetic and developmental origins.

Further, our results bring new light to the debate on the symmetry/asymmetry of facial expression muscles in humans and primates^22^,^23^. Recent developmental studies suggest that the left and right facial muscles separate from each other early in ontogeny: but in fact, the left muscles are actually ontogenetically more closely related to the base of the pulmonary trunk, and the right ones to the base of the aorta^24^. Also, functional studies in humans show that asymmetrical use of facial muscles is crucial to make complex facial expressions^25^. Furthermore, functional and anatomical studies of human facial expressions have shown that asymmetrical use of facial muscles is less prominent, and that innervations patterns of muscles are more symmetric, in the upper face (muscles located above the upper brow) than in the mid-face and lower face^26^,^27^. Since human speech tends to involve symmetrical muscle contraction, asymmetrical use of facial muscles is likely related to non-verbal communication in our own species. The phenotypic modules identified here place these developmental, functional, and anatomical observations in a completely new and quantitative context: contrary to expected bilateral orofacial muscular and musculoskeletal complexes, here we report the presence of left and right orofacial modules. This supports the ontogenetic separation of left and right facial muscles and the ability to asymmetrically contract or relax facial muscles, and thus strike more complex facial expressions in humans. In addition, AnNA recovered a single module including both the left and right ocular/upper face facial muscles, in line with previous studies showing that innervations patterns and use of muscles are more symmetric in the upper face. Future studies will lead us to apply AnNA specifically to muscles of facial expression among other primate and mammal species to investigate which anatomical structures may be unique to humans and which others have deeper evolutionary origins.

## Methods

### Anatomical dissection

We undertook anatomical dissections of 12 human cadavers (performed by RD) and an extensive literature review (also done by RD) to document the number and specific connections/attachments of all bones, cartilages, and muscles of the normal adult human head; details about these dissections, the individuals dissected, and all references reviewed in the literature review are given in Diogo & Wood's 2012 monograph about the comparative anatomy and evolution of human muscles^17^. The human cadavers were already stored (frozen) in RD's lab at the Department of Anatomy, Howard University College of Medicine (HUCM). Dissections took place in compliance with welfare guidelines approved by HUCM and the local ethics committee. After study, the cadavers were managed by HUCM according to US laws.

### Anatomical network modeling

We built an anatomical network model of the head's musculoskeletal system, which comprises all anatomical units of the human head, as well as the different types of physical interaction among them (Supplementary Data). For the purpose of this study, the skeletal and the muscular systems were analyzed separately–as two independent network models–in addition to the analysis carried out for the network model representing the entire musculoskeletal system of the head. Thus, we used different definitions of node and connection for each of these network models. The *skeletal network* comprises the bones and cartilage of the head and associated structures (skull, ear ossicles, mandible, neck cartilages, cervical vertebrae, and upper thoracic bones): nodes represent bones and connections represent physical articulations among them (sutural, synchondrosal, and synovial). The *muscular network* comprises the muscles of the head: nodes represent muscles and connections represent tendinous joints and fibrous fusions among them. The *musculoskeletal network* comprises all the above-mentioned anatomical parts of the head: nodes represent bones, cartilages, and muscles, and connections represent the above-described physical articulations, as well as fibrous, and tendinous attachments of muscles onto bones and cartilages. Network nodes were coded in and stored as *igraph* objects using the *igraph* package in *R*^28^.

### Identifying connectivity modules in musculoskeletal networks

A connectivity module is here defined as a group of anatomical units with more connections among them than to other units outside their group^3^. We identified the number and composition of connectivity for each anatomical network by maximizing the strength of modularity quantified as the modularity Q-value over all potential partitions^29^. We identified potential partitions in the musculoskeletal network using an heuristic method: first we performed a walk-trap algorithm of length 3 and then we resolved the best partition by taking the division that outputs the maximum Q value^30^. Q is the difference between the actual proportion of the connections within nodes in the same module and the expected proportion in a random network Q = Σ^M^(*e_mm_* – *a_m_*^2^), where *M* is the total number of modules, *e_mm_* is the proportion of links within module *m*, and *a_i_* is the proportion of links of nodes in *m*. Q ranges from −1 to 1: Q > 0 indicates that the number of the connections among elements within the same module are higher than expected at random. In networks with a significantly strong modular organization Q varies from 0.3 to 0.7, higher values being rare^29^. The identification of modules in anatomical networks was performed using the *igraph* package in *R*^28^.

### Labels

1 Occipital, 2 Parietal left, 3 Parietal right, 4 Temporal left, 5 Temporal right, 6 Sphenoid, 7 Zygomatic left, 8 Zygomatic right, 9 Frontal, 10 Ethmoidal, 11 Nasal left, 12 Nasal right, 13 Maxilla left, 14 Maxilla right, 15 Lacrimal left, 16 Lacrimal right, 17 Palatine left, 18 Palatine right, 19 Nasal concha left, 20 Nasal concha right, 21 Vomer, 22 Malleus left, 23 Malleus right, 24 Incus left, 25 Incus right, 26 Stapes left, 27 Stapes right, 28 Mandible, 29 Hyoid bone, 30 Thyroid cartilage, 31 Arytenoid cartilage left, 32 Arytenoid cartilage right, 33 Cricoid cartilage, 34 Sternum, 35 Clavicle left, 36 Clavicle right, 37 Scapula left, 38 Scapula right, 39–45 Cervical vertebrae 1 to 6, 46 Tensor tympani left, 47 Tensor tympani right, 48 Stapedius left, 49 Stapedius right, 50 Levator palpebrae superioris left, 51 Levator palpebrae superioris right, 52 Superior oblique left, 53 Superior oblique right, 54 Inferior oblique left, 55 Inferior oblique right, 56 Superior rectus left, 57 Superior rectus right, 58 Inferior rectus left, 59 Inferior rectus right, 60 Medial rectus left, 61 Medial rectus right, 62 Lateral rectus lefts, 63 Lateral rectus right, 64 Platsma left, 65 Platsma right, 66 Occipitalis left, 67 Occipitalis right, 68 Auricularis posterior left, 69 Auricularis posterior right, 70 Risorius left, 71 Risorius right, 72 Zygomaticus major left, 73 Zygomaticus major right, 74 Zygomaticus minor left, 75 Zygomaticus minor right, 76 Frontalis left, 77 Frontalis right, 78 Temporoparietalis left, 79 Temporoparietalis right, 80 Auricularis anterior left, 81 Auricularis anterior right, 82 Auricularis superior left, 83 Auricularis superior right, 84 Orbicularis oculi left, 85 Orbicularis oculi right, 86 Depressor supercilii left, 87 Depressor supercilii right, 88 Corrugator supercilii left, 89 Corrugator supercilii right, 90 Levator labii superioris alaeque nasi left, 91 Levator labii superioris alaeque nasi right, 92 Procerus left, 93 Procerus right, 94 Buccinatorius left, 95 Buccinatorius right, 96 Levator labii superioris left, 97 Levator labii superioris right, 98 Nasalis left, 99 Nasalis right, 100 Depressor septi nasi left, 101 Depressor septi nasi right, 102 Levator anguli oris facialis left, 103 Levator anguli oris facialis right, 104 Orbicularis oris left, 105 Orbicularis oris right, 106 Depressor labii inferioris left, 107 Depressor labii inferioris right, 108 Depressor anguli oris left, 109 Depressor anguli oris right, 110 Mentalis left, 111 Mentalis right, 112 Mylohyoideus left, 113 Mylohyoideus right, 114 Digastricus anterior left, 115 Digastricus anterior right, 116 Tensor veli palatini left, 117 Tensor veli palatini right, 118 Masseter left, 119 Masseter right, 120 Temporalis (main body) left, 121 Temporalis (main body) right, 122 Pterygoideus lateralis (pars. sup.) left, 123 Pterygoideus lateralis (pars. sup.) right, 124 Pterygoideus lateralis (pars. inf.) left, 125 Pterygoideus lateralis (pars. inf.) right, 126 Pterygoideus medialis left, 127 Pterygoideus medialis right, 128 Stylohyoideus left, 129 Stylohyoideus right, 130 Digastricus posterior left, 131 Digastricus posterior right, 132 Stylopharyngeus left, 133 Stylopharyngeus right, 134 Trapezius left, 135 Trapezius right, 136 Sternocleidomastoideus left, 137 Sternocleidomastoideus right, 138 Constrictor pharyngis medius left, 139 Constrictor pharyngis medius right, 140 Constrictor pharyngis inferior left, 141 Constrictor pharyngis inferior right, 142 Cricothyroideus left, 143 Cricothyroideus right, 144 Constrictor pharyngis superior left, 145 Constrictor pharyngis superior right, 146 Palatopharyngeus left, 147 Palatopharyngeus right, 148 Levator veli palatini left, 149 Levator veli palatini right, 150 Salpingopharyngeus left, 151 Salpingopharyngeus right, 152 Thyroarytenoideus left, 153 Thyroarytenoideus right, 154 Cricoarytenoideus lateralis left, 155 Cricoarytenoideus lateralis right, 156 Arytenoideus transversus left, 157 Arytenoideus transversus right, 158 Arytenoideus obliquus left, 159 Arytenoideus obliquus right, 160 Cricoarytenoideus posterior left, 161 Cricoarytenoideus posterior right, 162 Geniohyoideus left, 163 Geniohyoideus right, 164 Genioglossus left, 165 Genioglossus right, 166 Hyoglossus left, 167 Hyoglossus right, 168 Styloglossus left, 169 Styloglossus right, 170 Palatoglossus left, 171 Palatoglossus right, 172 Sternohyoideus left, 173 Sternohyoideus right, 174 Omohyoideus (pars. sup.) left, 175 Omohyoideus (pars. sup.) right, 176 Omohyoideus (pars. inf.) left, 177 Omohyoideus (pars. inf.) right, 178 Sternothryroideus left, 179 Sternothryroideus right, 180 Thyrohyoideus left, 181 Thyrohyoideus right.

## Supplementary Material

Supplementary Information Supplementary Information

## Figures and Tables

**Figure 1:**
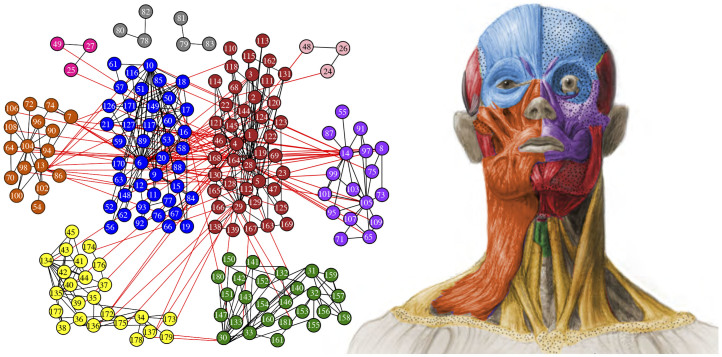
Modules of the human head identified using AnNA. In *red*, the lower jaw/inner ear complex; in *blue*, the mid/upper face complex; in *green*, the laryngeal complex; in *yellow*, the neck complex; in *orange and purple*, the oral/ocular complexes; in *light and dark grey*, the superficial ear complexes; and in *light and dark pink*, the inner ear complexes. Strength of modularity (Q-value) 0.5921. See labels in Methods. This figure was drawn by Christopher Smith.

**Figure 2:**
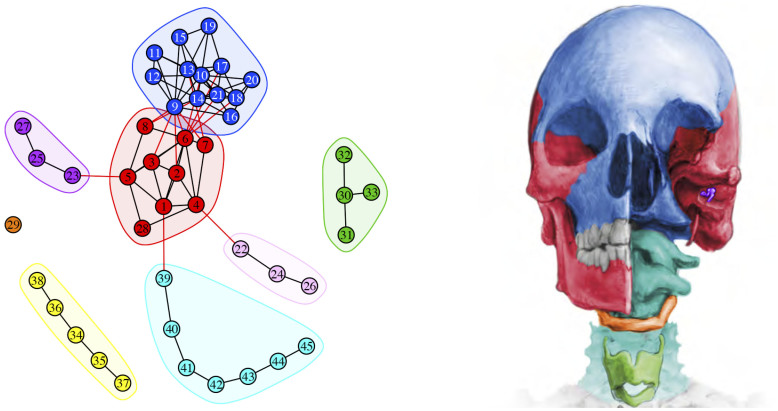
Modules of the head skeleton identified using AnNA. In *red*, the cranial complex; in *blue*, the facial complex; in *green*, the thyroid complex; in *yellow*, the thoracic complex; in *cyan*, the cervical complex; in *light and dark purple*, the ossicles complexes; and in *orange*, the hyoid one-bone module. Strength of modularity (Q-value) 0.4977. See labels in Methods. This figure was drawn by Christopher Smith.

**Figure 3:**
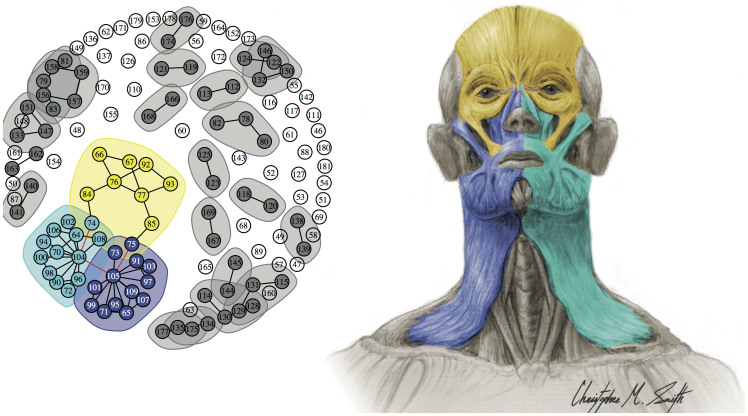
Modules of the head musculature identified using AnNA. In *yellow*, the ocular/upper face complex; in *light and dark blue*, the orofacial complexes; and in *grey*, the 21 smaller blocks of inter-connected muscles. In the absence of bones, most muscles are totally disconnected from the three major muscle modules (in *white*). Strength of modularity (Q-value) 0.8323. See labels in Methods. This figure was drawn by Christopher Smith.

**Table 1:** Phenotypic modules of the human head identified using AnNA

*Musculoskeletal Network*			
Modules	Bones/Cartilages	Muscles	Complex
**Module 1**	Hyoid, Malleus, Mandible, Occipital, Parietal, Temporal	Auricularis posterior, Constrictor pharyngis medius, Constrictor pharyngis superior, Digastricus anterior, Digastricus posterior, Geniohyoideus, Genioglossus, Hyoglossus, Masseter, Mentalis, Mylohyoideus, Pterygoideus lateralis inferior, Pterygoideus lateralis superior, Styloglossus, Stylohyoideus, Temporalis main body, Tensor tympani	*Lower jaw/inner ear*
**Module 2**	Ethmoid, Frontal, Lacrimal, Nasal concha, Nasal, Palatine, Sphenoid, Vomer	Corrugator supercilii, Frontalis, Inferior rectus, Lateral rectus, Levator veli palatini, Levator palpebrae superioris, Medial rectus, Occipitalis, Orbicularis oculi, Palatoglossus, Procerus, Pterygoideus medialis, Superior oblique, Superior rectus, Tensor veli palatini	*Mid/Upper face*
**Module 3**	Arytenoid, Cricoid, Hyoid, Thyroid	Arytenoideus obliquus, Arytenoideus transversus, Constrictor pharyngis inferior, Cricoarytenoideus lateralis, Cricoarytenoideus posterior, Cricothyroideus, Palatopharyngeus, Salpingopharyngeus, Stylopharyngeus, Thyroarytenoideus, Thyrohyoideus	*Laryngeal*
**Module 4**	Clavicle, Scapula, Sternum, Vertebrae	Omohyoideus inferior, Omohyoideus superior, Sternocleidomastoideus, Sternohyoideus, Sternothryroideus Trapezius	*Neck*
**Module 5 (left) & Module 6 (right)**	Maxilla, Zygomatic	Buccinatorius, Depressor anguli oris, Depressor labii inferioris, Depressor septi nasi, Depressor supercilii, Inferior oblique, Levator anguli oris facialis, Levator labii superioris alaeque nasi, Levator labii superioris, Nasalis, Orbicularis oris, Platysma myoides, Risorius, Zygomaticus major, Zygomaticus minor	*Oral/ocular*
**Module 7 (left) & Module 8 (right)**	*none*	Auricularis superior, Auricularis anterior, Temporoparietalis	*Superficial ear*
**Module 9 (left) & Module 10 (right)**	Incus, Stapes	Stapedius	*Inner ear*

*Skeletal Network*		
Modules	Bones/Cartilages	Complex
**Module 1**	Mandible, Occipital, Parietals, Sphenoid, Temporals, Zygomatics	*Cranial*
**Module 2**	Ethmoidal, Frontal, Lacrimals, Maxillae, Nasal conchae, Nasals, Palatines, Vomer	*Facial*
**Module 3**	Arytenoid, Cricoid, Thyroid	*Thyroid*
**Module 4**	Clavicles, Scapulae, Sternum	*Thoracic*
**Module 5**	Cervical vertebrae	*Cervical*
**Module 6 (left) & Module 7 (right)**	Incus, Malleus, Stapes	*Ossicles*
**Module 8**	Hyoid	*Hyoid*

*Muscular Network*		
Modules	Muscles	Complex
**Module 1**	Frontalis, Occipitalis, Orbicularis oculi, Procerus, Zygomaticus minor	*Ocular/upper face*
**Module 2 (left) & Module 3 (right)**	Buccinatorius, Levator labii superioris, Depressor anguli oris, Depressor labii inferioris, Levator anguli oris facialis, Levator labii superioris alaeque nasi, Nasalis, Depressor septi nasi, Orbicularis oris, Platsma myoides, Risorius, Zygomaticus major	*Orofacial*
