# Supplementary material for: Anatomical networks reveal the musculoskeletal modularity of the human head
Source: Sci Rep. 2015 Feb 6;5:8298. doi: 10.1038/srep08298 (PMC5389032; doi:10.1038/srep08298)
Supplement: Supplementary Information [file srep08298-s1.doc]

**Anatomical networks reveal the musculoskeletal modularity of the human head**

Borja Esteve-Altava^1*^, Rui Diogo^2*^, Christopher Smith^2^, Julia C. Boughner^3^, and Diego Rasskin-Gutman^1*^

1. Theoretical Biology Research Group, Cavanilles Institute of Biodiversity and Evolutionary Biology, University of Valencia, 46071 Valencia, Spain.

2. Department of Anatomy, Howard Univ. College of Medicine, Washington, DC, USA.

3. Department of Anatomy and Cell Biology, Univ. of Saskatchewan, Saskatoon, SK, Canada.

* Corresponding authors:

Borja Esteve-Altava: Telf. +34620435250. E-mail: boresal@gmail.com

Diego Rasskin-Gutman: Telf. +34963544463. E-mail: diego.rasskin@uv.es

Rui Diogo: Telf. +1202640982. E-mail: rui.diogo@howard.edu

**1. Supplementary Methods**

**Analysis of the head network architecture**. We have evaluated the whole architecture of each network model (skeletal, muscular and musculoskeletal) in order to identify differences in their fundamental pattern of organization. Specifically, for each network we have quantified the density of connections (D), the average clustering coefficient (C), the average shortest path length (L), the presence of the small-world phenomenon, and the presence of a hierarchical organization of connections:

D is the number of existing anatomical contacts with respect to the total maximum possible according to the total number of anatomical elements,
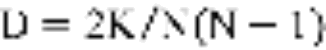
, where *K* is the number of connections in the network, and *N* the number of elements.

C is the arithmetic mean of the clustering coefficient of all anatomical elements in the network,
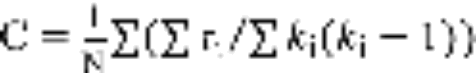
, where *τ_i_* is the number of triangular motifs including element *i*, and *k_i_* the number of connections of element *i*.

L is the arithmetic mean of the shortest path length between all pairs of elements in the network,
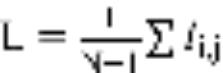
, where *l_i,j_* is the minimum number of connections that connects elements *i* and *j*.

The presence of the small-world phenomena is assessed by comparing the values of C and L of each anatomical network with those of 10,000 random equivalent networks, with the same number of elements and connections re-arranged randomly. After correcting for the network size, an empirical network is small-world if it fulfills the following condition:
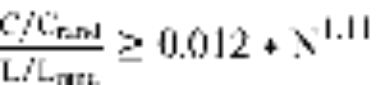
. The identification of connectivity modules in an anatomical network is linked to the presence of a small-world organization, in which parts are not connected regularly or totally at random, but with an organization in between ^31, 32^. Thus, connectivity modules in small-world, anatomical network are morphologically meaningful because they arise as a consequence of this characteristic organization, whereas regular networks are not modular and in random networks modules occur by chance.

The presence of a hierarchical organization of connections is assessed by fitting the connectivity distribution, P(*k*), and clustering coefficient distribution, C(*k*), of each network to a power-law ^33^. The P(*k*) is the probability to find an element with a given number of connections in the network:
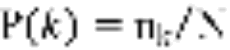
. Similarly, the C(*k*) measures the clustering coefficient mean of all elements with *k* connections:
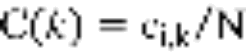
. In hierarchical networks both P(*k*) and C(*k*) are expected to fit a power-law distribution (e.g.,
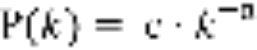
); in contrast, in scale-free networks only the P(*k*) fits a power-law, while in random networks none of these distributions fits a power-law ^33^. Hierarchical organizations emerge due to the presence of highly clustered groups of elements, which promotes the formation of an also hierarchical modular organization where connectivity modules can be grouped as sub-modules (or blocks) of higher-scale modules ^34, 35^.

Assessing the presence or absence of small-world and hierarchical features in empirical networks is important to interpret the outcomes of the modularity analysis. This requires comparing anatomical networks with randomly generated networks (null model), in which elements are connected with the same probability–that is, all connections are possible. Contacts among bones and muscles do not form by chance and not all contacts are possible (e.g., nasal with occipital), and the same is true for connections in many other types of biological networks such as genetic, ecological or social; however, comparing against randomness is a requisite to identify these two generic properties in all complex networks. In contrast, analyzing the biological processes underlying the actual connectivity patterns among anatomical parts demands other, more sophisticated null models to use as comparative baselines (as examples of this later use of network models see ^36, 37^). In the context of this study, comparing our empirical anatomical networks to random equivalent null models has the sole purpose to identify the presence of a small-world and a hierarchical organization.

**2. Supplementary Tables**

**Supplementary Table 1. Architecture of anatomical network models of the human head**

| **Network** | **D** | **C** | **L** | **Connected components** | **Small-world** | **Hierarchical** | **Q** |
| --- | --- | --- | --- | --- | --- | --- | --- |
| Skeletal | 0. 087 | 0.341 | 3.835 | 3 | YES | NO | 0.498 |
| Muscular | 0.008 | 0.503 | 3.007 | 3 | YES | YES | 0.832 |
| Musculoskeletal | 0.025 | 0.482 | 3.616 | 1 | YES | YES | 0.592 |

D, density of connections; C, average clustering coefficient; L, average shortest path length; Q, strength of modularity.

**3. Supplementary Discussion**

**Architecture of the human head**. The skeletal, muscular, and musculo-skeletal networks of the human head show some differences in their overall organization of connections (Supplementary Table 1 and Supplementary Fig. 1). The skeletal network comprises 45 bone/cartilage connected among them by 86 contacts (sutures, synchondroses, and synovial joints). The skeletal network has a small-world organization as previously reported for the human skull, which excluded ear ossicles and mandible; however, the skeletal network here does not show a hierarchical organization of connections. The muscular network comprises 136 muscles sparsely connected by 78 contacts (fiber fusions and well-defined tendons). Although the muscular network shows parameter values that indicate it is a small-world, hierarchical network, this result might be biased by their high disconnectedness among most muscles in the absence of a head skeleton. Most of its muscles are disconnected from the three principal connected components or totally disconnected from any other muscle in absence of bones. Finally, the musculoskeletal network comprises 181 elements (including bones and muscles) connected by 412 contacts. This network consists of one connected component that shows a small-world, hierarchical organization of connections.

**Supplementary Figure 1. Degree distribution and clustering coefficient distribution of the skeletal, muscular, and musculo-skeletal networks.** **A**) P(*k*) of the skeletal network (α = 0.714, r^2^ = 0.385). **B**) C(*k*) of the skeletal network (α = 0.712, r^2^ = 0.92). **C**) P(*k*) of the muscular network (α = 1.318, r^2^ = 0.876). **D**) C(*k*) of the muscular network (α = 1.154, r^2^ = 0.999). **E**) P(*k*) of the musculo-skeletal network (α = 1.175, r^2^ = 0.702). **F**) C(*k*) of the musculo-skeletal network (α = 0.903, r^2^ = 0.713).

**Supplementary References**

1. Watts, DJ & Strogatz, SH. Collective dynamics of ‘smallworld’ networks. *Nature* **393**, 440-442 (1998).
2. Humphries, MD & Gurney, K. Network ‘small-world-ness’: A quantitative method for determining canonical network equivalence. *PLoS ONE* **3**, e0002051 (2008).
3. Barabási, A-L, Oltvai, ZN. Network biology: understanding the cell's functional organization. *Nat Rev Genet* **5**, 101-13 (2004).
4. Ravasz, E, et al. Hierarchical organization of modularity in metabolic networks. *Science* **297**, 1551-1555 (2002).
5. Mason, O & Verwoerd, M. Graph theory and networks in Biology. *IET Syst Biol* **1**, 89-119 (2007).
6. Esteve-Altava, B & Rasskin-Gutman, D. Beyond the functional matrix hypothesis: A network null model of human skull growth for the formation of bone articulations. *J Anat* **225**, 306-316 (2014).
7. Esteve-Altava, B & Rasskin-Gutman, D. Theoretical morphology of tetrapod skull networks. *C R Palevol* **13**, 41-50 (2014).
